# Guhong Injection Protects Against Apoptosis in Cerebral Ischemia by Maintaining Cerebral Microvasculature and Mitochondrial Integrity Through the PI3K/AKT Pathway

**DOI:** 10.3389/fphar.2021.650983

**Published:** 2021-05-13

**Authors:** Huifen Zhou, Yu He, Jiaqi Zhu, Xiaojie Lin, Juan Chen, Chongyu Shao, Haitong Wan, Jiehong Yang

**Affiliations:** ^1^ Institute of Cardiovascular-Cranial Disease, School of Life Sciences, Zhejiang Chinese Medical University, Hangzhou, China; ^2^ College of Pharmacy, Zhejiang Chinese Medical University, Hangzhou, China; ^3^ College of Basic Medical Science, Zhejiang Chinese Medical University, Hangzhou, China

**Keywords:** Guhong injection, ischemic stroke, cerebral microvascular, mitochondria, antiapoptosis, PI3K/Akt pathway

## Abstract

Guhong injection (GHI) can be used for the treatment of ischemic stroke. We investigated the antiapoptotic activity of GHI, its ability to repair the cerebral microvessels and mitochondria, and the PI3K/AKT signaling pathway of GHI against cerebral ischemia. Western blot and immunohistochemical analyses were used to determine the expression of cleaved caspase-3, B-cell lymphoma-2 (Bcl-2), cytochrome c (Cyt-c), basic fibroblast growth factor (BFGF), vascular endothelial growth factor (VEGF), transforming growth factor-β1 (TGF-β1), and proteins in the PI3K/AKT signaling pathway. Transmission electron microscopy and scanning electron microscopy were used to evaluate the structures of the cerebral microvasculature and cells. Hoechst 33342 staining was used to evaluate the nuclear morphology. FITC-AV/PI double staining was used to measure the antiapoptotic effects. The fluorescent dye JC-1 was used to measure mitochondrial membrane potential. The enzyme-linked immunosorbent assay (ELISA) was used to detect the activities of matrix metalloproteinase-9 (MMP-9). Biochemical assay kits were used to detect the activities of lactate dehydrogenase (LDH), superoxide dismutase (SOD), and malondialdehyde (MDA). Compared with the middle cerebral artery occlusion (MCAO) group, there was decreased infarct volume and significantly improved neurological deficits in the GHI group. In addition, the expression of Bcl-2 was significantly upregulated, while the expression of Cyt-c, Bax, and cleaved caspase-3 was notably downregulated. GHI administration attenuated the pathological change and morphology of the cerebral microvasculature, and immunohistochemical staining indicated that the expressions of BFGF, VEGF, and TGF-β1 were significantly increased. The cell morphology, cell viability, cell nuclei characteristics, and mitochondrial morphology normalized following GHI treatment, which decreased the release of Cyt-c and the mitochondrial membrane potential. The levels of LDH, MMP-9, and MDA decreased, while SOD increased. Moreover, GHI administration inhibited the activation of the PI3K/AKT signaling pathway in rat brain microvascular endothelial cells (rBMECs) following oxygen/glucose deprivation (OGD) injury. Therefore, our results show that GHI administration resulted in antiapoptosis of cerebral cells and repair of cerebral microvessels and mitochondria *via* the PI3K/AKT signaling pathway.

## Introduction

Ischemic stroke is currently the world’s leading cause of death and contributes to an enormous social and economic burden (Palm et al. 2016; Zhou et al., 2019). Tissue plasminogen activator (tPA), the only FDA-approved acute stroke medication, has a limited therapeutic time window and severe complications (Liu et al., 2018; Machado-Pereira et al., 2018; Hughes et al., 2020). The causes of ischemic stroke are complex. Western medicine only utilizes a single target in cases of ischemic stroke, and the therapeutic effect is limited. In contrast, multiple targets are used in traditional Chinese medicine (TCM), and obvious beneficial effects are achieved for ischemic injury (Sun et al., 2015).

Guhong injection (GHI), a new compound preparation, has been used in the treatment of ischemic stroke for a long time in China. It is composed of aceglutamide and safflower extract. It was reported that aceglutamide decreases motor dysfunction and delays neuronal death following ischemia, which is associated with the inhibition of proapoptotic factor TRAF1 (Zhang et al., 2015). Prior studies have reported that the main component of safflower extract is hydroxysafflower yellow A (HSYA), which has antiapoptotic, antithrombotic, and anti-inflammatory effects. According to our previous works, compared with the HSYA monomer, HSYA in combination with aceglutamide possesses a synergetic effect of antiapoptosis and anti-inflammation that acts to protect against cerebral ischemic injury (Deng et al., 2018). The AUC of HSYA for GHI administered to rats increased significantly (Chen et al., 2016). GHI exerted a strong and ameliorative anti-inflammatory effect on cerebral ischemic injury (Ai et al., 2017), and satisfactory clinical manifestations without side effects were observed in cases of stroke (Xiaowei et al., 2014). Overall, these previous studies indicated that GHI might play a protective role and could be a new potential treatment for stroke. However, the protective effects of GHI induced by brain ischemic injury have not yet been elucidated in detail.

After cerebral ischemic injury, the PI3K/AKT pathway is activated and involved in cell proliferation, differentiation (Zhou et al., 2020), angiogenesis (Chen et al., 2019), and survival (Beker et al., 2019). The activation of the PI3K/AKT signaling pathway is related to the increase in many growth-stimulating factors, angiogenic molecules, and vascular endothelial growth factor. The PI3K/AKT signaling pathway is widely distributed in cells and plays a key role in the regulation of apoptosis. Mitochondria also play an important role in the process of apoptosis (Faulkner et al., 2020). The mitochondrion is not only the receptor of the endogenous apoptotic pathway (Zhang et al., 2020) but is also the amplifier of the apoptotic signal that facilitates the process of apoptosis so that it can proceed quickly and efficiently (Shoshan-Barmatz et al., 2020). In this study, we investigated the efficacy of GHI against cerebral ischemic injury *in vitro* and *in vivo*, and explored its potential mechanism by evaluating the associated proteins of antiapoptosis, the cerebral microvasculature, and mitochondrial integrity *via* the PI3K/AKT pathway.

## Materials and Methods

### Reagents

GHI was provided by Tonghua Guhong Pharmaceutical Co., Ltd. (Batch NO.: 170322). Dulbecco’s modified Eagle’s medium (DMEM-F12) was obtained from GIBCO (Invitrogen Corporation, CA, United States). Fetal bovine serum (FBS) was purchased from Hyclone (Thermo Scientific, MA, United States). EDTA and gelatin were obtained from Genview (Gen-view Scientific Inc., TX, United States). Collagenase type II and bovine serum albumin (BSA) were obtained from Sigma (Sigma-Aldrich Co. LLC, MO, United States). Trypsin (1:250) was purchased from Amresco (United States). Fluorescein isothiocyanate (FITC)-goat anti-rabbit IgG antibody (II antibody) and rabbit antihuman factor VIII antibody (I antibody) were purchased from Sino-American Biotechnology Company (China). 3-(4, 5-dimethylthiazol-2-yl)-2, 5-diphenyl-tetrazolium bromide (MTT) was purchased from Sigma-Aldrich (St. Louis, MO, United States). The fluorescent kit for Hoechst 33342 was purchased from Beyotime Institute of Biotechnology Co., Ltd. (Hangzhou, China). A FITC Annexin V apoptosis detection kit and JC-1 were obtained from BD Biosciences (CA, United States). Kits for the determination of lactate dehydrogenase (LDH), matrix metalloproteinase-9 (MMP-9), superoxide dismutase (SOD), and malondialchehyche (MDA) were obtained from the Jiancheng Bioengineering Institute (Nanjing, China). Anti-Bax (ab32503), anti–Bcl-2 (ab32124), and mouse monoclonal anti-ATP synthase β-chain (mitochondria) (ab170947) antibodies were obtained from Abcam Co. (Abcam, Cambridge, MA, United States). DAPI, anti-Akt, anti–p-Akt, anti–caspase-3, anti-cleaved caspase-3, rabbit monoclonal anti–cytochrome c, and anti–β-actin antibodies were purchased from Cell Signaling Technology, Inc. (CST, Danvers, MA, United States). 2, 3, 5-triphenyl tetrazoliumchloride (TTC) was purchased from Sigma-Aldrich Co. (St. Louis, United States).

### Quality Control of GHI

Each 1 ml of GHI contains aceglutamide equivalent to 30 mg and safflower equivalent to 0.5 g. According to the statement of the National Drugs Surveillance administrative bureau standard, the quality control standard of GHI is that the content of aceglutamide (C_7_H_12_N_2_O_4_) should be in the range of 27–33 mg/ml and the content of HYSA (C_27_H_32_O_16_) should not be lower than 0.15 mg/ml when detected by HPLC (Supplementary Figure S1). The HPLC fingerprinting and its quality control of GHI were performed by using an established method in our lab (Ai et al., 2017).

### Animal

Forty healthy adult male Sprague–Dawley (SD) rats, weighing 280 ± 10 g, were obtained from the Central Animal Facility of Zhejiang Chinese Medical University (Laboratory animal certificate: SCXK:2018-0012) and were housed under a 12/12-h dark/light cycle and specific pathogen-free (SPF) conditions (temperature 22–24°C and humidity 50–60%), respectively, and the animals were given standard diet and water *ad libitum* for the duration of the study for 7 days so that they could adapt to the experimental environment. The methods were carried out in accordance with international guidelines on the ethical use of animals (Mason and Matthews 2012).

### Animal Model and Grouping

The rats underwent intraluminal occlusion using monofilament for the preparation of the transient middle cerebral artery occlusion (tMCAO) model (Longa et al., 1989). All the rats were anesthetized by intraperitoneal (i.p.) injection of pentobarbital sodium (2%, 2 ml/kg) after weighing. The right common carotid artery (CCA), the external carotid artery (ECA), and the internal carotid artery (ICA) were exposed. A 4–0 monofilament nylon suture with a rounded tip was carefully inserted into the ICA *via* the ECA. Reperfusion was performed 1 h after tMCAO. To ensure the establishment of the model, the regional cerebral blood flow was monitored using a laser-Doppler flowmeter. Surgery was performed on the rats of the sham group without inserting the nylon suture.

The rats were randomly divided into five groups: the sham group (Sham group), the ischemia-reperfusion group (I/R group), the GHI 2.5 ml/kg group, the GHI 5 ml/kg group, and the GHI 10 ml/kg group (the doses were chosen based on clinical medication conversion and the results of preliminary animal experiments), with eight rats in each group for the TTC staining study, three rats in each group for the molecular biological study, and three rats in each group for the pathological study. The total number of rats was 70. All groups received intraperitoneal injections twice per day, at scheduled times, for seven consecutive days. Normal saline (10 ml/kg/d) was administered to the rats in the Sham and I/R groups.

### Neurological Function Evaluation

According to Garcia’s method (Garcia et al., 1995), neurological defect was graded on a scale of 3–18 at day 1 and day 7 after reperfusion in response to tMCAO. The score given to each rat was a composite of eight individual test scores, including spontaneous activity, climbing, forepaw outstretching, symmetry in the movement of the four limbs, body proprioception, and response to the touch of vibrissae.

### Measure of Infarct Size

The rats were euthanized after day 7. The brains were removed, sliced into 2-mm coronal slices starting at 1 mm from the frontal pole, and then immediately incubated in 2% 2,3,5-triphenyltetrazolium chloride (TTC) at 37°C for 15 min. Then, the brain slices were scanned using a flat-bed scanner, and infarct volume was quantified with a computerized image analyzer. The results of infarct volume are presented as a percentage of the total volume of slices.

### Culture of Rat Brain Microvascular Endothelial Cells and the Oxygen/Glucose Deprivation Model

Based on previous methods (Liu et al., 2013; Liao et al., 2018), cerebral gray matter from male Sprague–Dawley (SD) rats (50–60 g, purchased from the Animal Center of Nanjing University) was chopped and passed through 200-μm and 77-μm meshes. The tissue remaining on the 77-μm mesh screen was collected and digested with 0.1% collagen type II for 20 min at 37°C, followed by resuspension in 25% BSA and centrifugation. The precipitate acquired was incubated in Dulbecco’s modified Eagle’s medium/Nutrient Mixture F-12 (DMEM/F12) medium supplemented with 20% FBS, 3 mg/ml glucose, 0.58 mg/ml l-glutamine, 100 U/ml penicillin, and 100 mg/ml streptomycin, and then seeded into a gelatin-coated culture flask at a density of 1 × 10^5^/ml and cultured at 37°C in an incubator. Staining of cells for factor VIII was observed by using a fluorescence microscope, and the growth curve was recorded. We employed rBMECs at the third passage, which gained 98% purity in the present study.

According to a previous study (Li et al., 2012), cells were in ischemic condition with OGD in glucose- and sodium pyruvate–free DMEM (supplemented without FBS) flow and were exposed to the environment (94% N_2_, 5% CO_2_, and 1% O_2_) for 6 h. The normal group cells were incubated while being exposed to normal oxygen conditions for 6 h.

### Groups and Treatment

The safe concentrations of GHI were determined by the 3-(4,5-dimethylthiazol-2-yl) (MTT) test. Briefly, rBMECs were cultured in 96-well plates, the designated concentration of GHI was added, and the cells were cultured for 6 h. Based on the MTT method, we obtained the optical density (OD) values from the microplate reader at a wavelength of 490 nm. The results revealed that 0–110 μL/ml was safe, with cell viabilities being greater than 90%, and therefore, we selected 25, 50, and 100 μl/ml as the different concentrations for further research. Then, the rBMECs were randomly labeled under five groups, including the control group, the OGD group (incubated under OGD for 6 h without pretreatments), and three groups treated with different concentrations of GHI (pretreated with GHI in three different concentrations before culture under OGD for 6 h). As for the Western blot assays of Bcl-2, caspase-3, Bax, Akt, and p-Akt, the OGD + LY 294002 group (pretreated with 20 μmol/L of LY 294002 for 1 h of cultivation and then incubated under OGD for 6 h) and the GHI 100 μL/ml + LY 294002 group (pretreated with 20 μmol/L of LY 294002 for 1 h of incubation and then cultured with 100 μL/ml of GHI under OGD for 6 h) were set up, along with the control group, the OGD group, and three groups with varying concentrations of GHI, which were administered as above.

### Cell Morphological Changes and Viability Assay

After 6 h of incubation with various preparations, an optical microscope (Leica, Wetzlar, Germany) equipped with a camera was used to observe the cell morphological changes. Cells (1×10^4^ cells/well) were seeded in 96-well plates and then incubated at 37°C. The cells were processed under OGD conditions according to the different groups. After experimental interventions, the MTT assay was performed by adding 5 mg/ml MTT solution to each well, and the plates were incubated for 6 h at 37°C. Then, the cell viability was measured by evaluating the absorbance at the wavelength of 490 nm.

### Hoechst 33342 Apoptotic Staining

Under OGD conditions, rBMECs in the different groups were treated, washed in cold PBS, fixed in 10% neutral buffered formalin for 10 min, and then washed again in cold PBS. Finally, the cells were stained with Hoechst 33342 (2 μg/ml) for 20 min at 4°C in the dark and then captured under a fluorescent microscope.

### Flow Cytometric Apoptosis Assay

First, rBMECs were gathered into flow cytometry tubes, washed with cold PBS, and then centrifuged and resuspended twice. After that, the cells were cultured with Annexin V and propidium iodide (PI) in the dark for 30 min at room temperature. Subsequently, rBMECs were resuspended with binding buffer and analyzed by flow cytometry.

### Mitochondrial Membrane Potential Assay

The fluorescent dye JC-1 was used to determine the mitochondrial membrane potential (Δψ). The cells were suspended for 10 min in an equal amount of JC-1 staining solution (5 μg/ml) at 37°C. At least 10,000 cells were collected from each sample, and JC-1 fluorescence was analyzed by flow cytometry.

### Transmission Electron Microscopy and Scanning Electron Microscopy

The rat brain was perfused for 40 min with a fixative made of 4% formaldehyde and 2% glutaraldehyde in 0.1 mol/L phosphate buffer. For transmission electron microscopy, a coronal slice in the cortex penumbra area of approximately 1 mm thick was taken. The slice was placed in freshly prepared 3% glutaraldehyde overnight at 4°C. After rinsing with phosphate buffer three times, the tissue block was postfixed in 1% osmium tetroxide in 0.1 mol/L phosphate buffer for 2 h at 4°C. The samples were dehydrated and embedded in Epon 812. Ultrathin sections of cortex were stained with uranium acetate and lead citrate and examined using a transmission electron microscope (H-7650, Rili, Japan). For scanning electron microscopy, the samples were cut into blocks and placed in freshly prepared glutaraldehyde for 2 h, rinsed with phosphate buffer, and then postfixed in 1% osmium tetroxide in 0.1 mol/L phosphate buffer for 2 h at 4°C. The samples were processed as routing and examined using a scanning electron microscope (SU8010, Rili, Japan).

The mitochondrial structure of rBMECs was observed using a TECNAI 10 transmission electron microscope (Philips, Holland). rBMECs were collected by centrifugation and fixed with a 1% (w/v) solution of osmium tetroxide. The samples were embedded in Epon 812-Araldite and cut into 50-nm-thick sections for observation.

### Immunocytochemistry Analysis

At day 7 after reperfusion from tMCAO, immunohistochemistry for basic fibroblast growth factor (BFGF), vascular endothelial growth factor (VEGF), and transforming growth factor-β1 (TGF-β1) was carried out following formalin fixing and paraffin embedding of sections. After dehydration in graded ethanol and transparency treatment with xylene, the brain block was embedded in paraffin and cut into 3-μm coronal sections on a rotary microtome (Microm HM340E, Germany). For immunohistochemical analysis, the sections were then incubated with primary antibody (rabbit-anti-rat, 1:100) overnight at 4°C after specific antigen was blocked with normal goat serum. The sections were washed and then incubated with the secondary antibody (goat anti-rabbit, 1:500). The stained sections were observed under a fluorescence microscope. Furthermore, the integrated optical density (IOD) of BFGF, VEGF, and TGF-β1 was measured by using Image-ProPlus 6.0 software (Media Cybernetics, United States) to average five microscopic fields (magnification 200×).

rBMECs (1×10^4^ cells/well) grown in a laser confocal glass substrate culture dish were fixed in 4% paraformaldehyde for 20 min at room temperature. For mitochondrial and cytochrome *c* staining, cells were blocked for 60 min with 5% normal goat serum in PBS containing 0.1% Tween 20 (PBST). After the blocking reaction, the cells were incubated with mouse monoclonal anti-ATP synthase β-chain (mitochondria) (1:50) and rabbit monoclonal anti–cytochrome *c* (anti–Cyt-c) (1:100) overnight at 4°C. The cells were washed in PBST three times for 5 min each time, and then soaked for 90 min in secondary antibody dilution buffer with the corresponding secondary antibodies: goat anti-mouse IgG-Alexa Fluor 594 (red; 1:1000) and goat anti-rabbit IgG-Alexa Fluor 488 (green; 1:1000). The cells were washed three times for 5 min each time in PBST and incubated for 5 min with 1 μg/ml 4′,6-diamidino-2-phenylindole (DAPI). Finally, the cells were washed five times for 5 min each time in PBST and three times for 5 min each time in PBS, and then subsequently embedded in mounting medium. Immunofluorescent images were visualized using Carl Zeiss Microscopy GmbH (Zeiss). Control experiments were performed with the omission of the primary antibodies, yielding negative results.

### Measurement of LDH, MMP-9, SOD, and MDA Levels

Another batch of the culture media was collected to detect the activities of lactate dehydrogenase (LDH) and matrix metalloproteinase-9 (MMP-9), and rBMECs were collected to measure the activities of superoxide dismutase (SOD) and malondialdehyde (MDA) in the different groups. The levels of MMP-9 were measured using commercially available enzyme-linked immunoassay kits, in accordance with the respective manufacturer’s instructions. The activities of LDH and SOD and the levels of MDA were measured using biochemical assay kits according to the manufacturer’s instructions.

### Western Blotting Analysis

Western blotting was conducted as previously reported. Proteins were separated using sodium dodecyl sulfate (SDS)-polyacrylamide gel and transferred to polyvinylidene fluoride (PVDF) membranes. The PVDF membrane was incubated with the following primary antibodies at 4°C overnight: Bcl-2 (1:500), Bax (1:500), cytochrome *c* (1:1,000), cleaved caspase-3 (1:1,000), p-Akt (1:1,000), Akt (1:1,000), caspase-3 (1:1,000), and β-actin (1:1,000). The PVDF membrane was then incubated with peroxidase-conjugated secondary antibodies for 2 h at room temperature. The gel images were obtained with ECL-Plus reagent (GE Healthcare, Piscataway, NJ, United States), and the results were normalized to β-actin.

### Statistical Analysis

Statistical analyses were carried out using SPSS 19.0 software. The data are expressed as the mean ± standard deviation. One-way analysis of variance (ANOVA) was used to assess the differences between groups, and then, Tukey’s test was performed. *p* < 0.05 was considered to have statistical significance.

## Results

### Effects of GHI on the Neurological Deficit in Focal Cerebral I/R Rats

**FIGURE 1:**
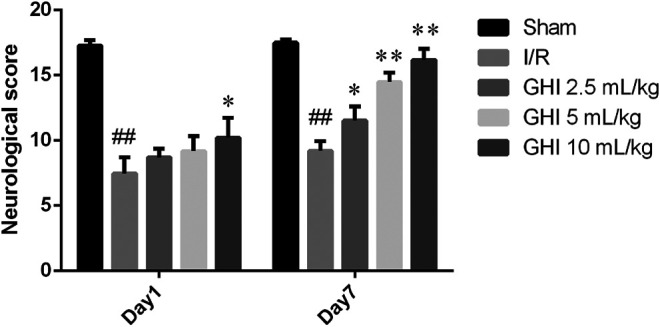
Effect of GHI on neurological deficit scores in focal cerebral I/R rats. Groups were as follows: Sham group (Sham), ischemia-reperfusion group (I/R group), GHI 2.5 ml/kg group, GHI 5 ml/kg group, and GHI 10 ml/kg group at 1 and 7 days, respectively. Values were expressed as mean ± SD (n = 8). ^##^ *p* < 0.01 vs. the Sham group, *p < 0.05, **p < 0.01 vs. the I/R group.

There were significant differences in neurological deficit scores among the Sham group, the I/R group, and the treated groups, as illustrated in Figure 1. After tMCAO, the neurological deficit score of the I/R group was significantly lower than that of the sham group (*p* < 0.01) at day 1 and day 7. Additionally, the scores in the GHI groups 7 days after reperfusion were significantly higher at day 1 than those of the I/R group (*p* < 0.01 or *p* < 0.05). These results suggest that the treatment of GHI improved neurological function recovery.

### Effect of GHI on Cerebral Infarct Volume in Focal Cerebral I/R Rats

**FIGURE 2:**
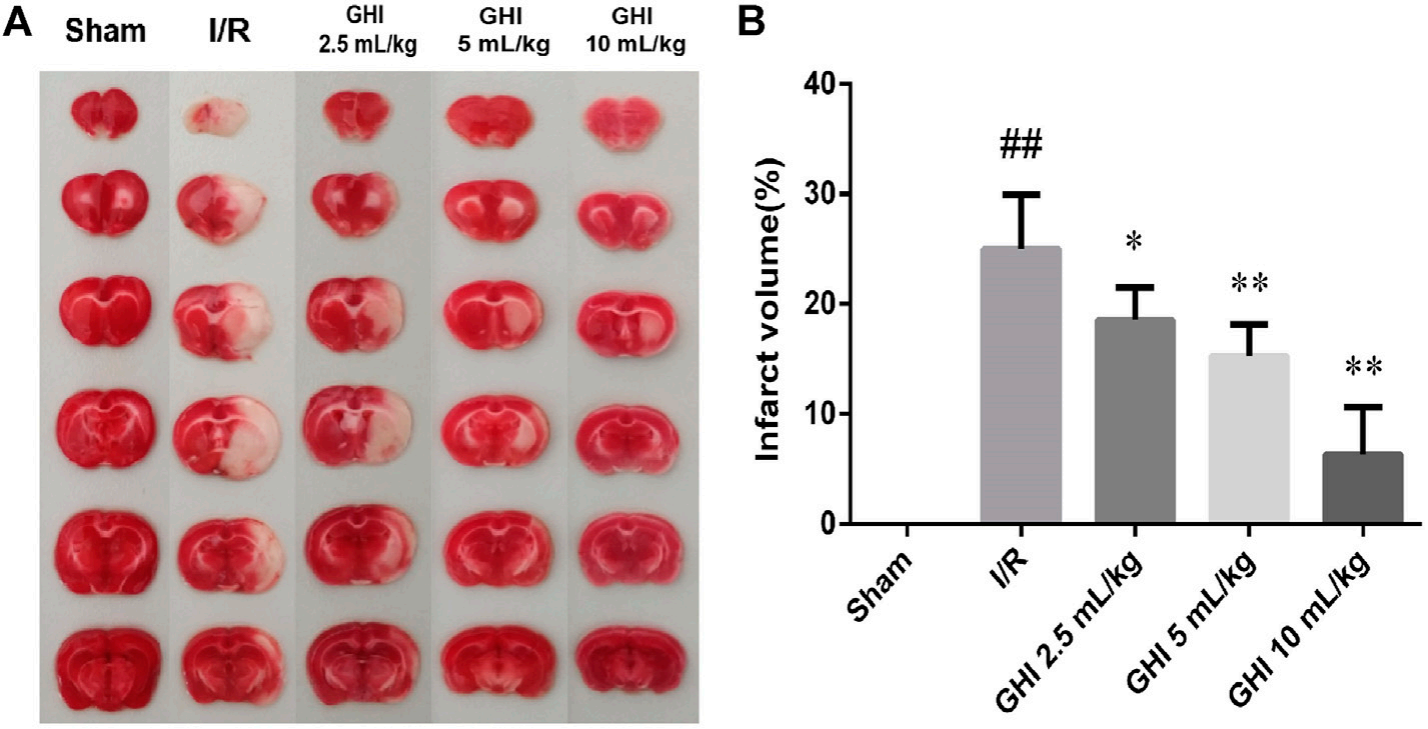
Effect of GHI on cerebral infarct volume in the focal cerebral I/R rats. Infarct volume was assessed by TTC staining at day 7 after tMCAO. **(A)** Representative TTC staining of the cerebral infarct of rat brain. **(B)** Infarct volumes assessed by TTC staining. Groups were as follows: Sham group (Sham), ischemia-reperfusion group (I/R group), GHI 2.5 ml/kg group, GHI 5 ml/kg group, and GHI 10 ml/kg group. The data were expressed as means ± SD (n = 8). ^##^ *p* < 0.01 vs. the Sham group, *p < 0.05, **p < 0.01 vs. the I/R group.

As shown in Figure 2, viable tissue was stained deep red by TTC staining, whereas the color of the area of infarction in the right cerebral hemisphere was white. The infarct volume in the I/R group was significantly increased in comparison to that in the Sham group (*p* < 0.01). The 100 μL/ml GHI group showed the smallest infarct volume (*p* < 0.01) when compared with the I/R group. These results suggest that GHI administration decreased the cerebral infarct volume.

### Effects of GHI on Protein Expression of Cleaved Caspase-3, Bcl-2, Bax, and Cyt-c in Focal Cerebral I/R Rats

**FIGURE 3:**
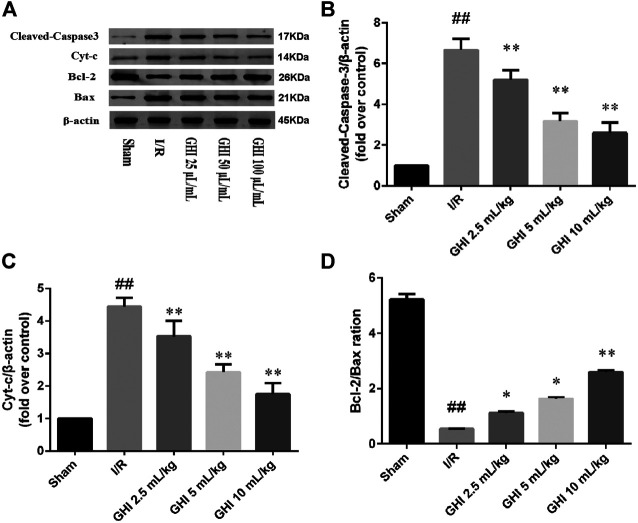
Western blot analysis of cleaved caspase-3, Bcl-2, Bax, and Cyt-c in the cerebral I/R region. Groups were as follows: Sham group (Sham), ischemia-reperfusion group (I/R group), GHI 2.5 ml/kg group, GHI 5 ml/kg group, and GHI 10 ml/kg group. ^##^ *p* < 0.01 vs. the Sham group, *p < 0.05, **p < 0.01 vs. the I/R group.

As shown in Figure 3, the Western blot assay demonstrated that cleaved caspase-3 and Cyt-c protein levels in the I/R group were higher than those in the Sham group at day 7 (*p* < 0.01). Compared with the I/R group, the cleaved caspase-3 and Cyt-c protein levels in the GHI groups were decreased in a dose-dependent manner (*p* < 0.01). Furthermore, compared with the Sham group, the Bcl-2/Bax ratio was remarkably decreased in the I/R group (*p* < 0.05). However, compared with the I/R group, the Bcl-2/Bax ratio in the GHI groups was notably enhanced in a dose-dependent manner (*p* < 0.01 or *p* < 0.05).

### Effects of GHI on the Cerebral Microvasculature in Focal Cerebral I/R Rats

**FIGURE 4:**
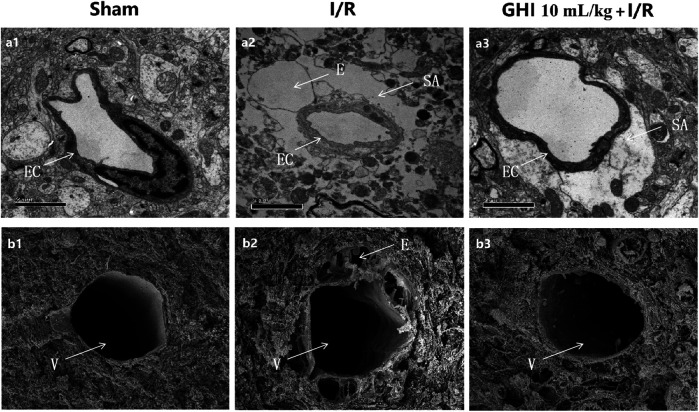
Ultrastructure of microvessels in the cerebral cortex. The transmission electron micrographs of the cerebral cortex are displayed in a1–a3; the scanning electron micrographs of the cerebral cortex are displayed in b1–b3. a1, b1: Sham group; a2, b2: I/R group; a3, b3: GHI 10 ml/kg + I/R group. E: perivascular edema; EC: endothelial cell; SA: swelling astroglial process; and V: venules (bar = 50 μm).

Transmission electron microscopy clearly identified the structures of the cerebral microvasculature in the cortex, as shown in Figure 4. Compared with the Sham group, a narrowed lumen, rough inner surface, evident swelling of the perivascular astrocyte end feet, and intracellular organelles that were frequently absent or scarce were observed in the I/R group, and these defects were apparently alleviated by GHI treatment. Further examination was performed using scanning electron microscopy under different experimental conditions. In accordance with the results observed by transmission electron microscopy, compared with the Sham group, I/R injury challenge caused capillary shrinkage with a rough endothelial surface, numerous pox-like projections into the lumen, and hypertrophy of the digitations of endothelial cell contacts. Perivascular vacuoles were often observed, which is a morphological manifestation consistent with brain edema. These abnormalities were decreased in rats treated with 10 ml/kg GHI following I/R injury.

### Effects of GHI on the Expression of BFGF, VEGF, and TGF-β1 in Focal Cerebral I/R Rats

**FIGURE 5:**
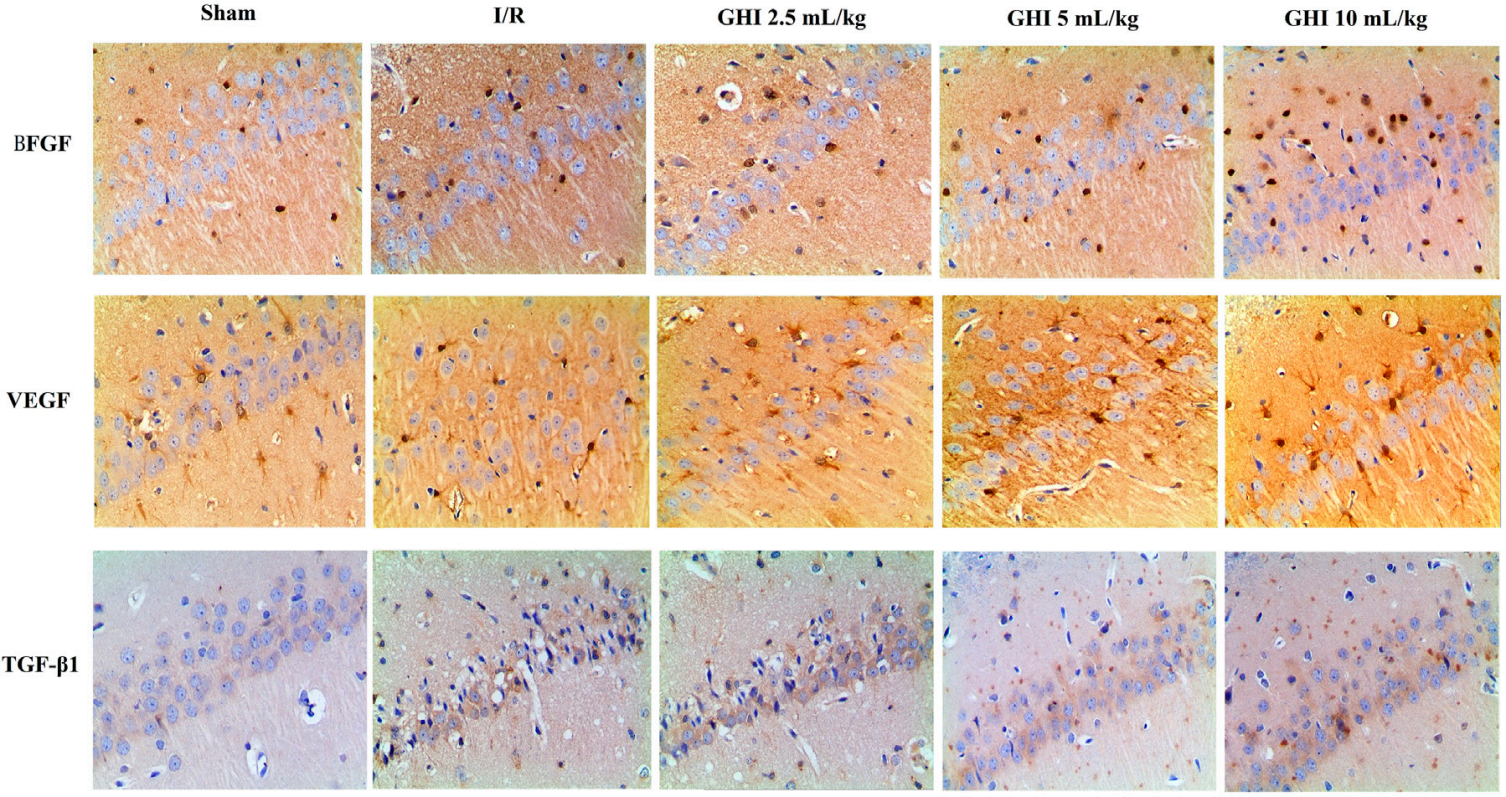
Expressions of BFGF, VEGF, and TGF-β1 immunostained tissue of focal cerebral I/R rats (magnification 200×). Groups were as follows: Sham group (Sham), ischemia-reperfusion group (I/R group), GHI 2.5 ml/kg group, GHI 5 ml/kg group, and GHI 10 ml/kg group.

**FIGURE 6:**
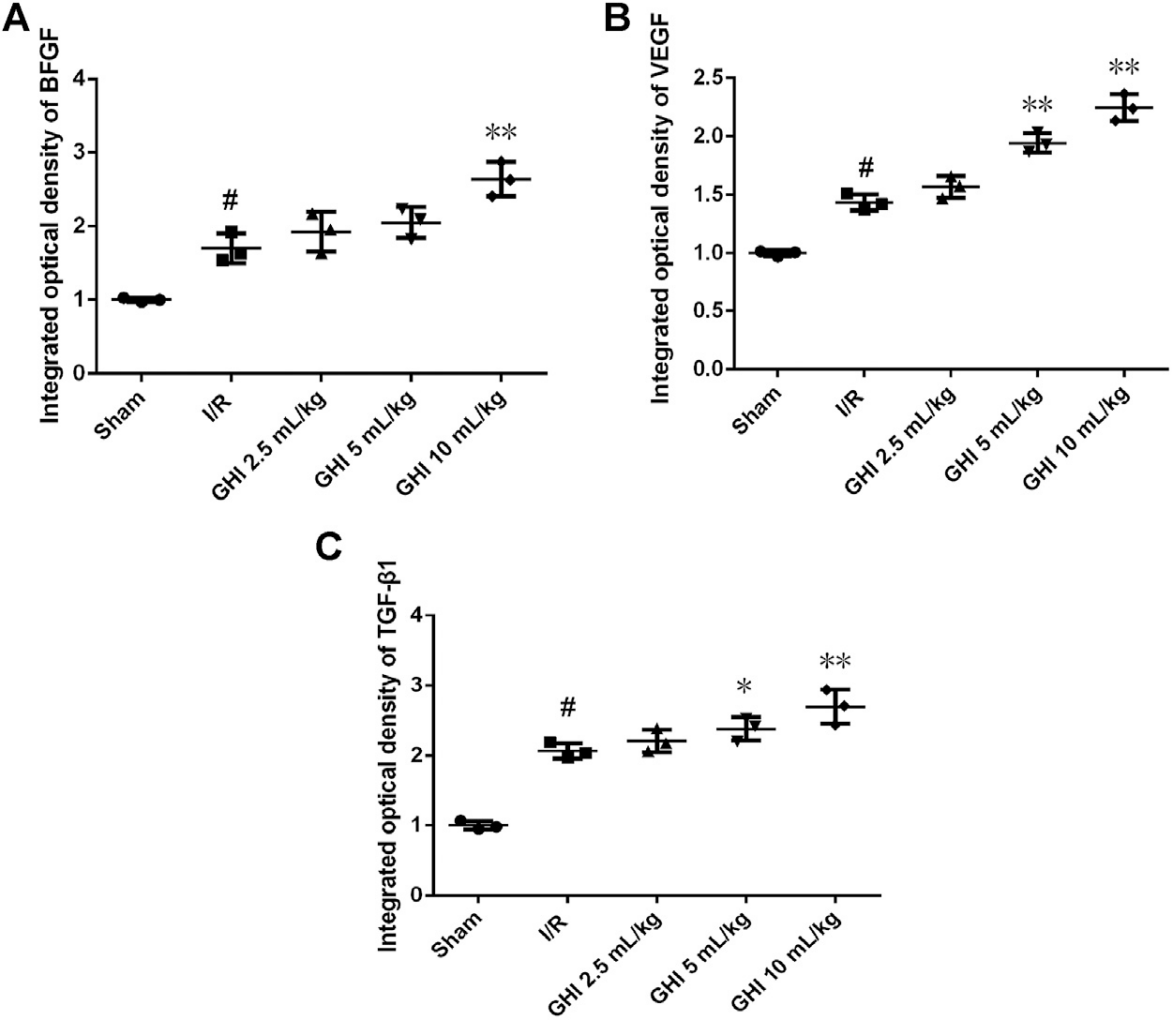
Immunohistochemical analysis of BFGF, VEGF, and TGF-β1 cerebral I/R hippocampal CA1 region. Groups were as follows: Sham group (Sham), ischemia-reperfusion group (I/R group), GHI 2.5 ml/kg group, GHI 5 ml/kg group, and GHI 10 ml/kg group. The data were expressed as means ± SD (n = 3 rats per group). ^##^ *p* < 0.01 vs. the Sham group, *p < 0.05, **p < 0.01 vs. the I/R group.

Immunohistochemical determination of BFGF, VEGF, and TGF-β1 was performed to determine whether GHI can promote angiogenesis. As shown in Figures 5, 6, the expression of BFGF, VEGF, and TGF-β1 increased significantly (all *p* < 0.01) in the cerebral I/R hippocampal CA1 region. Compared with the I/R group, the expression of BFGF, VEGF, and TGF-β1 in the GHI groups increased significantly (*p* < 0.01 or *p* < 005).

### Characteristics of rBMECs

**FIGURE 7:**
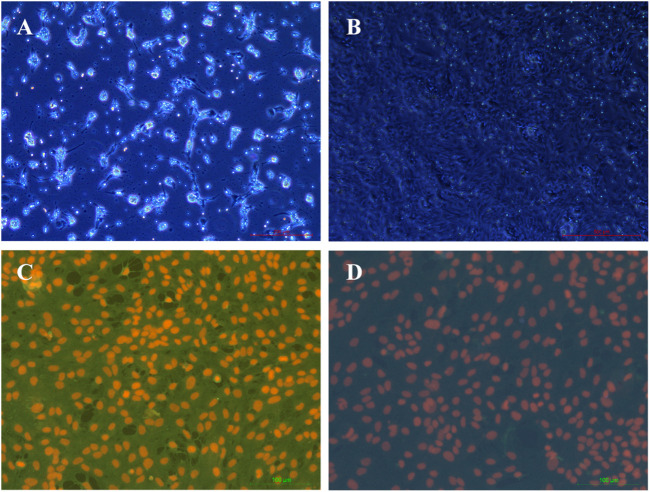
Characterization of rBMECs under a microscope. **(A)** rBMECs at 2 d, number of microvascular segments that appeared like a string of beads, and the wall of the tube was smooth and clear, **(B)** confluent subcultured cells formed cobblestoned morphology, **(C)** staining by factor VIII of cells, and **(D)** negative control.

In the initial passage, rBMECs grew mainly from clusters of microvascular fragments in a radial fashion with whirlpools and threads (Figure 7A). However, the dispersed cells presented a uniform spindle-like morphology and a cobblestone-like structure under confluence (Figure 7B). Their strongly positive immunostaining for factor VIII is widely considered to be the most reliable marker of endothelial cell-specific protein expression (Figures 7C,D). rBMECs were identified using fluorescence double labeling methods. The cell nuclei of the negative control only exhibited red fluorescence upon staining by PI, rather than green fluorescence. The samples treated with factor VIII and secondary antibody were scanned under the same parameters, and the cytoplasm displayed strong green fluorescence, indicating that the cells were considered to be rBMECs containing factor VIII. Nearly 100% of the cells were rBMECs through cell statistical counting.

### Effects of GHI on OGD-Induced Changes in Cell Morphology and Viability

**FIGURE 8:**
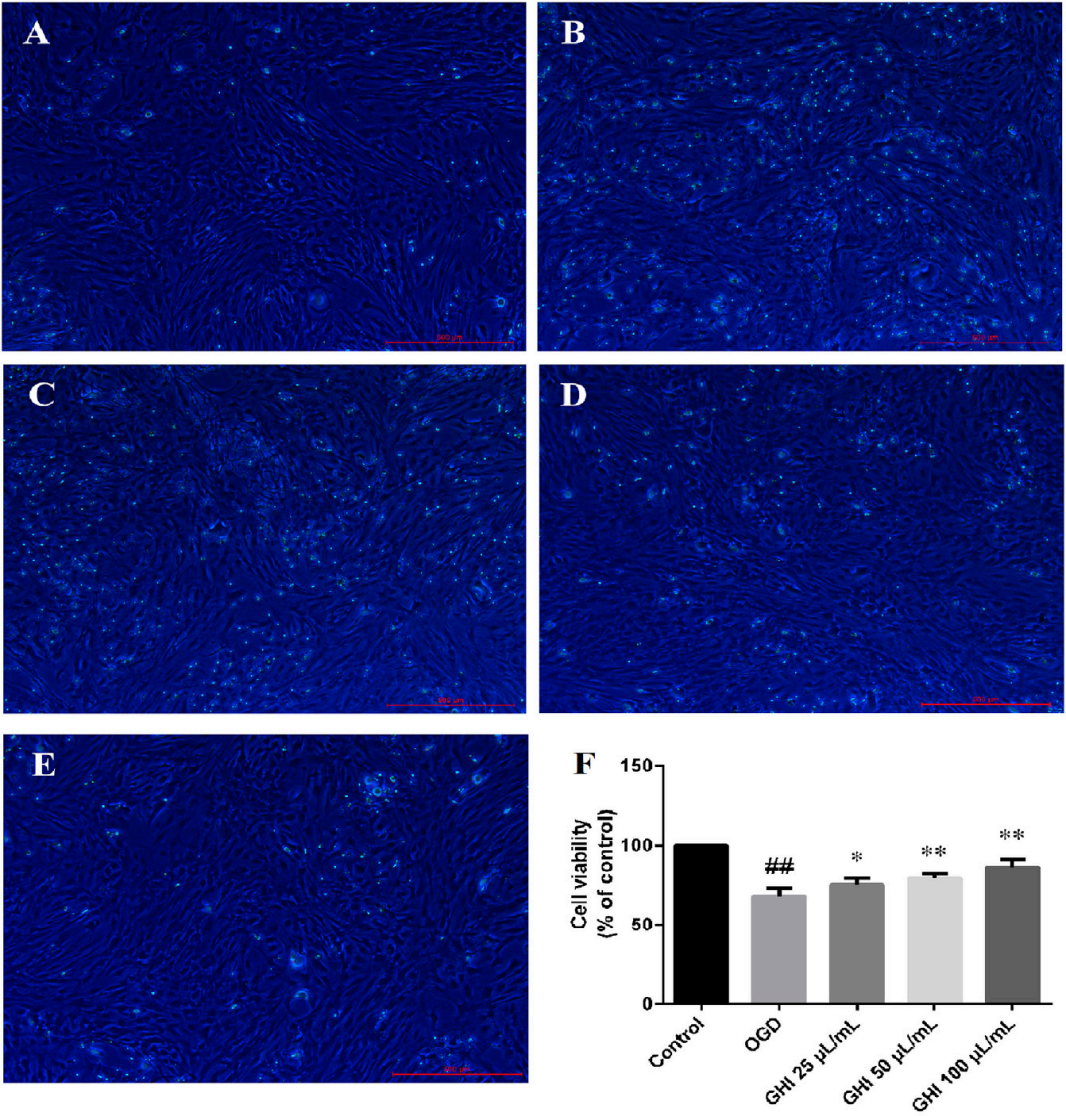
Effects of GHI on OGD-induced changes in cell morphology and viability (n = 6). **(A)** Control group, **(B)** OGD group, **(C)** GHI 25 μL/ml, **(E)** GHI 100 μL/ml, and **(F)** cell viability. ^##^ *P* < 0.01 compared with the Control group; *p < 0.05, **p < 0.01 compared with the OGD group.

Compared with untreated cells, rBMECs exposed to OGD for 6 h exhibited swelling, and the viability of rBMECs in the OGD group significantly decreased. Cell morphology was improved by pretreatment with 25, 50, or 100 μl GHI (Figures 8A–E). The viability of rBMECs significantly decreased following OGD injury. However, compared with the OGD group, the viability of rBMECs increased to 73.4 ± 4.2, 79.8 ± 2.6, and 86.0 ± 5.3% after pretreatment with 25, 50, and 100 μl/ml GHI, respectively (Figure 8F).

### Hoechst 33342 Staining Indicates that GHI Protected the Characteristics of rBMEC Nuclei Under the OGD Model

**FIGURE 9:**
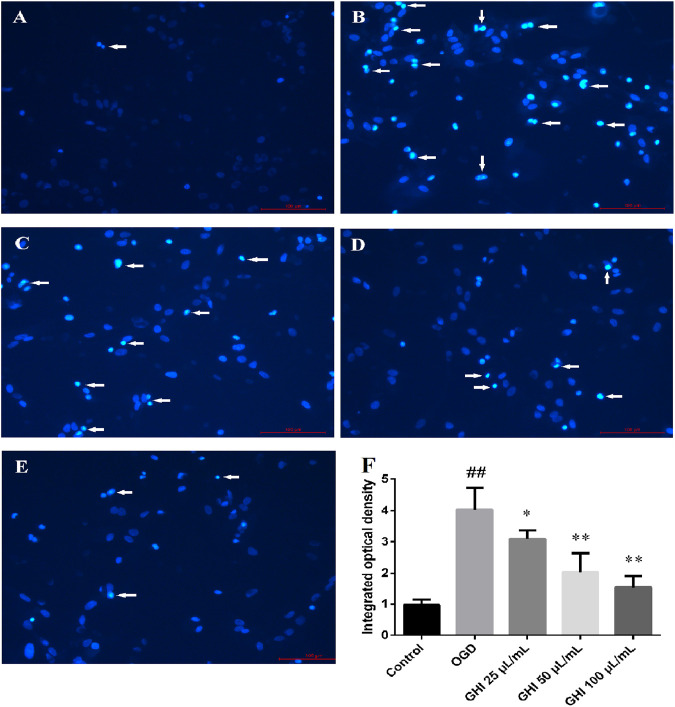
Effects of GHI on histochemical characterizations of rBMECs in OGD injury (n = 6). Arrowheads in the pictures indicate the nuclei of apoptosis cells. **(A)** Control group, **(B)** OGD group, **(C)** GHI 25 μL/ml, **(D)** GHI 50 μL/ml, **(E)** GHI 100 μL/ml, and **(F)** integrated optical density. ^##^ *P* < 0.01 compared with the control group; *p < 0.05, **p < 0.01 compared with the OGD group.

The nuclear morphologies of rBMECs in the five groups were examined by Hoechst 33342 staining due to its membrane permeability. As shown in Figure 9, OGD induced large changes in the nuclear morphology of rBMECs, including chromatin condensation and increased nucleus size. Compared with the control group, bright light staining showed that apoptosis significantly increased in the OGD group. There was remarkable improvement in the morphological changes of the nuclei induced by OGD in the GHI groups. This revealed that GHI decreased the apoptosis of rBMECs and protected against OGD-induced injury in a dose-dependent manner.

### The Antiapoptotic Effects of GHI on rBMECs with OGD Injury as Measured by FITC-AV/PI Double Staining

**FIGURE 10:**
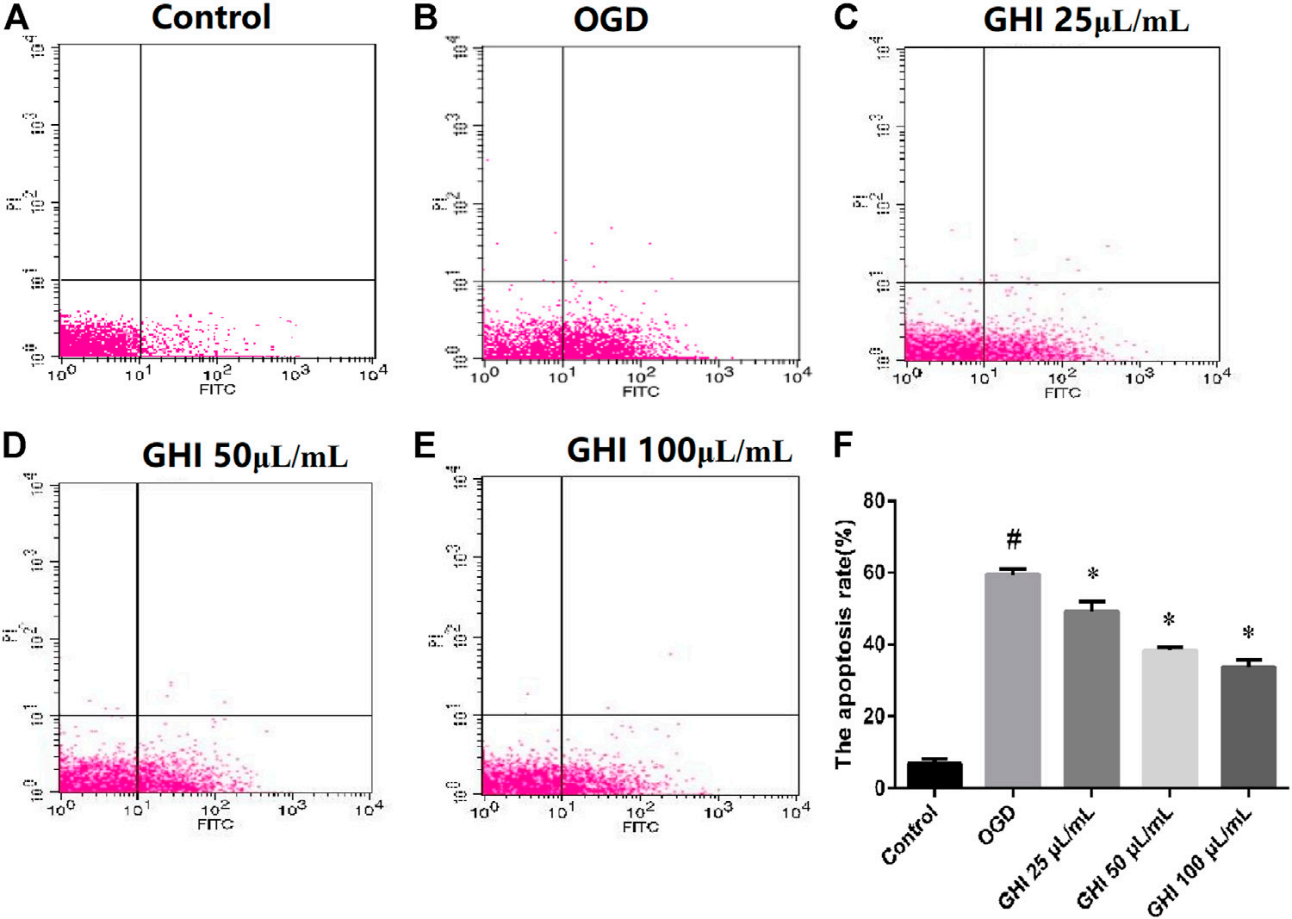
Effects of GHI on apoptosis rates of rBMECs in OGD injury. Values were expressed as the means ± SD (n = 3). **(A–E)** Flow cytometry images and **(F)** statistical analysis of the apoptosis rate. ^#^ *p* < 0.01 compared with the control group; *p < 0.01 compared with the OGD group.

To analyze the possible antiapoptotic ability of GHI under OGD, a FITC-AV/PI double staining method was used to measure the apoptosis rates of GHI by flow cytometry. As shown in Figure 10, compared with the Control group, the rBMEC apoptosis rate significantly increased in the OGD group (*p* < 0.01). Compared with the OGD group, there were significant decreases in the apoptosis rates of rBMECs at the early stage (*p* < 0.01) in the GHI groups, and the data indicated that the most optimal effect was observed in the GHI 100 μL/ml group.

### Effects of GHI on the Mitochondrial Membrane Potential of rBMECs Under OGD Injury

**FIGURE 11:**
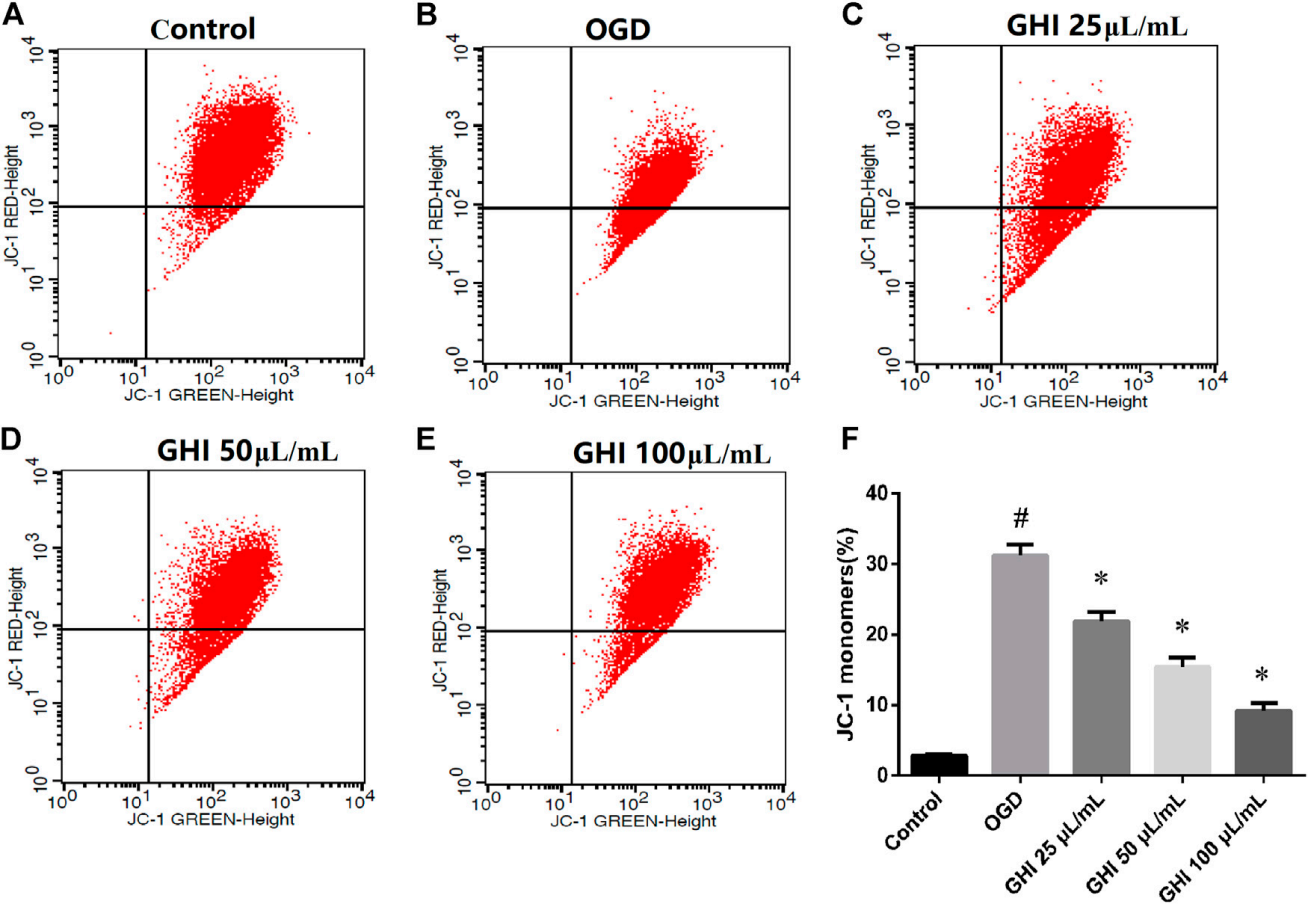
GHI treatment decreases the proportion of the JC-1 monomer of rBMECs under OGD injury (n = 3). **(A–E)** Flow cytometry images and **(F)** statistical analysis of JC-1. ^#^ *p* < 0.01 compared with the control group; *p < 0.01 compared with the OGD group.

The loss of mitochondrial membrane potential suggests that apoptosis can be detected by using a unique fluorescent cationic dye, JC-1. It has been revealed that the decrease in the red–green fluorescence intensity ratio is a marker of mitochondrial depolarization. As shown in Figure 11, the proportion of the JC-1 monomer significantly increased due to OGD injury in comparison to that in the control group. Compared with the OGD group, the proportion of the JC-1 monomer was significantly decreased in the GHI groups in a dose-dependent manner.

### Effects of GHI on the Mitochondrial Ultrastructure of rBMECs Under OGD Injury

**FIGURE 12:**
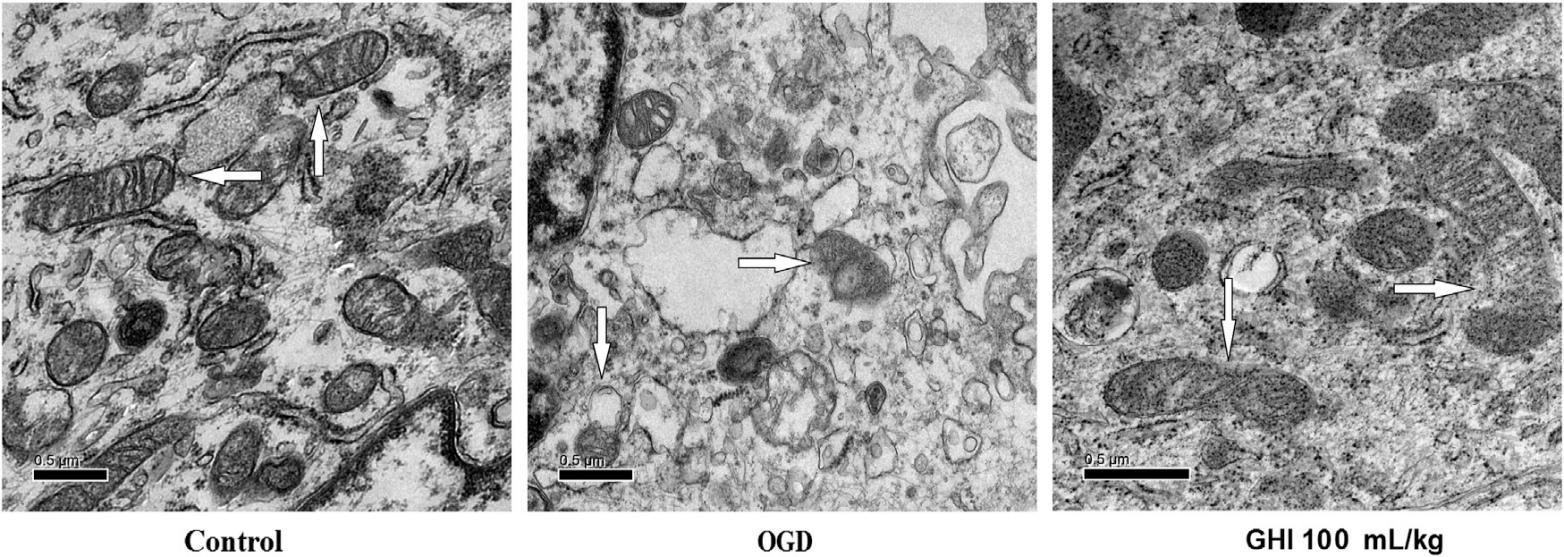
Evaluation of the mitochondrial ultrastructure by electron microscopy (n = 3).

As shown in Figure 12, the ultrastructure of rBMEC mitochondria was observed by transmission electron microscopy at 6 h after OGD. Compared with the control group, the rBMECs exhibited typical apoptotic markers after OGD injury, especially mitochondrial changes consisting of incomplete and unclear cristae. However, there was only mild injury of rBMECs in the GHI 100 mL/ml treatment group, with significant differences from the control group. These results suggest that GHI administration might play a protective role by maintaining the stability of mitochondrial morphology during OGD injury.

### Effects of GHI on the Release of Cyt-C from Mitochondria After OGD-Induced Injury

**FIGURE 13:**
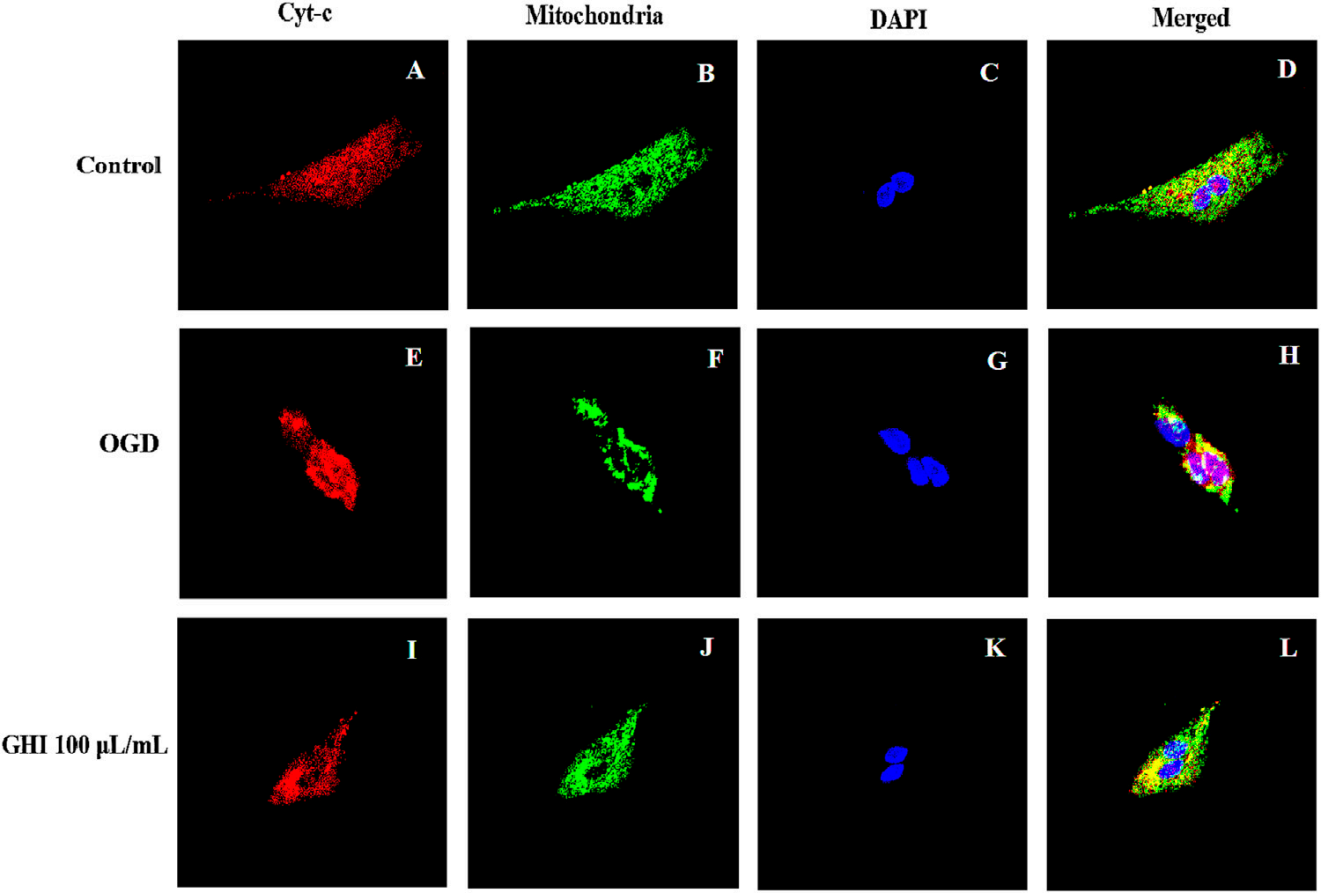
Effects of GHI on the release of Cyt-c from mitochondria after OGD-induced injury. GHI decreases the release of Cyt-c from mitochondria. Cyt-c **(A,E,I)**, mitochondria **(B,F,J)**, and DAPI **(C,G,K)** staining revealed an apparent maintained within the mitochondria under OGD conditions when treated with 100 μl/ml of GHI **(I–L)** compared to the without GHI. Cyt-c and mitochondria double-positive cells are shown in panels: **(D,H,L)**. Red: Cyt-c, blue: DAPI, and green: mitochondria (n = 3).

As shown in Figure 13, Cyt-c was retained in mitochondria under normal oxygen conditions (Figures 13A–D). However, under hypoxic conditions, Cyt-c was released from mitochondria (Figures 13E–H). After GHI 100 μl/ml treatment, there was less release of Cyt-c (Figures 13I–L).

### Effects of GHI on the Levels of LDH, MMP-9, SOD, and MDA in rBMECs After OGD-Induced Injury

**FIGURE 14:**
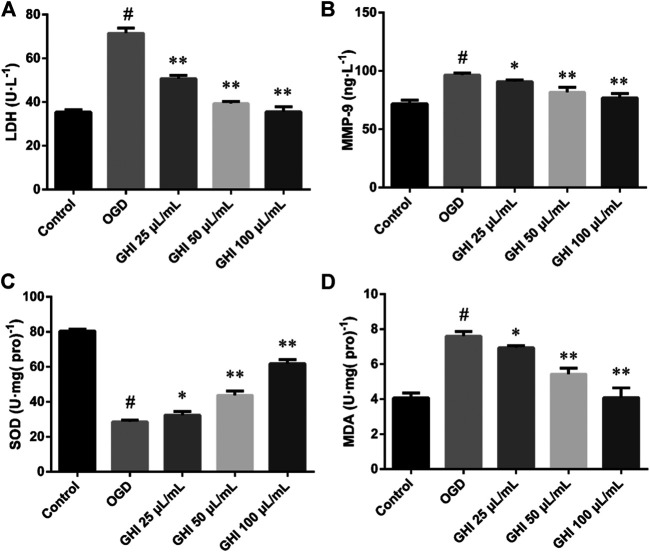
Effects of GHI on the levels of LDH, MMP-9, SOD, and MDA in rBMECs after OGD-induced injury. The data were expressed as means ± SD (n = 8). ^#^ *p* < 0.01 compared with the Control group; *p < 0.05, **p < 0.01 compared with the OGD group.

As demonstrated in Figure 14, compared with the control group, there were substantial increases in the levels of LDH, MMP-9, and MDA, and decreases in SOD levels in the OGD group (*p* < 0.01). However, after treatment with GHI, the activities of LDH, MMP-9, and MDA were extraordinarily lower than those in the OGD group (*p* < 0.01), and the activity of SOD was extraordinarily higher than that in the OGD group (*p* < 0.01). These results suggest that GHI can significantly reduce OGD-induced injury.

### Effects of GHI on the Expression of Apoptosis-Related Proteins in rBMECs After OGD-Induced Injury

**FIGURE 15:**
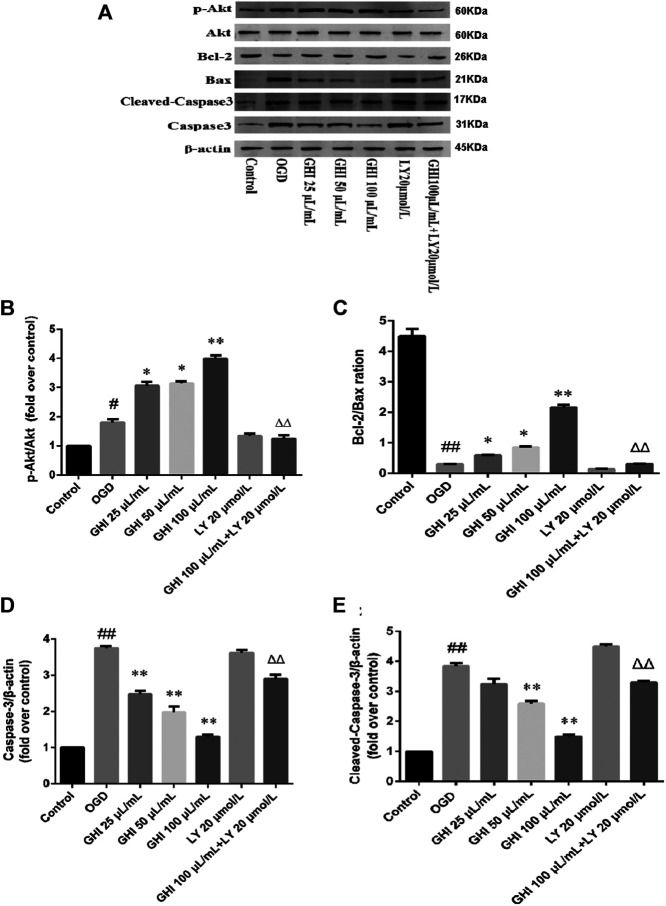
GHI attenuated OGD-induced injury by regulating the PI3K/Akt pathway. **(A)** Representative immunoblots of Akt, p-Akt, Bax, Bcl-2, cleaved caspase-3, and caspase-3 proteins. **(B)** The ratio of p-Akt/Akt. **(C)** The ratio of Bcl-2/Bax. **(D)** The levels of caspase-3. **(E)** The levels of cleaved caspase-3. Data are shown as the mean ± SD (n = 3). ^#^ *p* < 0.05, ^##^ *p* < 0.01 compared with the Control group; ^*^ *p* < 0.05, ^**^ *p* < 0.01 compared with the OGD group, ^Δ^ *p* < 0.05, ^ΔΔ^ *p* < 0.01 compared with the GHI 100 μL/ml group.

To perform an in-depth evaluation of the cascade of apoptosis after treatment with GHI, the changes in apoptosis-related proteins were determined by Western blot. To determine the mechanism of GHI, we used PI3K/Akt inhibitor LY 294002. The results showed that OGD did not change the expression level of total Akt, but phosphorylated Akt (p-Akt) was increased in the OGD group, and it was further enhanced by GHI in a dose-dependent manner. LY 294002 blocked GHI-increased p-Akt/Akt ratios (Figures 15A,B).

To further examine the mechanism GHI uses to decrease apoptosis, we determined the expression of Bax, Bcl-2, cleaved caspase-3, and caspase-3 in rBMECs. Bax and Bcl-2 may play a major role in determining the survival or death of cells after apoptotic stimulation. Figure 15C depicts a decrease in the Bcl-2/Bax ratio in the OGD group, and this decrease was greatly blocked by GHI in a dose-dependent manner. Caspase-3 is an important component of the final pathway leading to the occurrence of cell apoptosis. As illustrated in Figures 15D,E, OGD resulted in a noticeable increase in caspase-3 and cleaved caspase-3 expression, while GHI reduced the level of caspase-3 and cleaved caspase-3 expression compared with that of the OGD group in a dose-dependent manner. However, compared with the GHI (100 μL/ml) group, treatment with GHI (100 μL/ml) + LY 294002 (20 μmol/L) markedly decreased the Bcl-2/Bax ratio and increased active caspase-3 and cleaved caspase-3 expression (Figures 15A,D,E). These results demonstrated that GHI reduced the apoptosis of rBMECs *via* OGD, probably through the PI3K/Akt pathway.

## Discussion

The current study provides evidence that GHI exerted a protective and therapeutic effect in a dose-dependent manner on focal cerebral ischemia in rats in regard to the improvement of neurological deficits and microvascular morphology, the reduction of cerebral infarction and brain tissue damage, and the function of inhibiting apoptosis and increasing vascular regeneration. Furthermore, we also observed that GHI not only ameliorated damaged cell morphology and loss of viability but also reduced mitochondrial damage and Cyt-c release. Notably, GHI was involved in the inhibition of apoptosis through the PI3K/Akt pathway in rBMECs following OGD.

The PI3K/Akt signaling pathway is widely distributed in cells and plays an important role in the regulation of apoptosis (Guo et al., 2019). Studies have reported that the activation of PI3K/AKT signaling is associated with increased levels of various growth-stimulating factors and pro-angiogenic molecules, such as matrix metalloproteinases (MMPs) and vascular endothelial growth factor (VEGF) (Lee et al., 2020). Basic fibroblast growth factor (BFGF), an important mitogenic factor, plays a key role in angiogenesis (Debreczeni et al., 2021). BFGF exerts its biological activities by binding to four structurally similar fibroblast growth factor receptors (FGFRs), especially FGFR1, in the presence of heparin (Rajendran et al., 2018). Ligand-induced receptor dimerization increases tyrosine kinase activity, thereby triggering the BFGF signaling cascade (PI3K/AKT) (Genet et al., 2019). VEGF is a specific polypeptide protein that promotes endothelial growth and angiogenesis (Yu et al., 2019). There is interplay between VEGF (A, B, C, D, and E) ligands and VEGF receptors (VEGFR-1, 2, and 3) (Colombo et al., 2018). Activation of VEGFR, a member of the receptor tyrosine kinase (RTK) family, will then lead to upregulation of multiple cellular pathways (PI3K/AKT) that promote cell growth and angiogenesis (Feng et al., 2019; Wu et al., 2019; Kaufman et al., 2021). Transforming growth factor-β1 (TGF-β1) is a polypeptide growth factor that can activate the PI3K/Akt signaling pathway by binding to TβR-I and TβR-II on fibroblasts and other cell membranes and activating receptors (Li et al., 2012; Liu et al., 2019). TGF-β1 induces the p85 subunit of PI3K to be recruited into focal adhesion kinase (FAK) (Vallée and Lecarpentier 2019). PI3K activates Akt by phosphorylation at the thr308 and ser473 sites (Lontay et al., 2020). Moreover, TGF-β1 activates Akt signaling through p38 mitogen–activated protein kinase (MAPK) and FAK (Choi et al., 2019). The occurrence of microvascular injury is presented following cerebral ischemia reperfusion (Thiankhaw et al., 2021).

In the present study, we found that GHI administration regulated the angiogenesis-related protein expression of BFGF, VEGF, and TGF-β1, compared with that in the I/R group. At the same time, these data showed that all angiogenesis-related parameters were significantly enhanced after GHI treatment. Growth factors, which include BFGF, VEGF, and TGF-β1, are involved in cell proliferation, cell cycling, and cell apoptosis (Kong et al., 2019). VEGF and BFGF enhance vascular formation, which promotes the expression of platelet-derived growth factor (PDGF)-related protein (Zhou et al., 2019). In addition, TGF-β1 was shown to be neuroprotective following the generation of MCAO (Abdel-Rahman et al., 2020). Angiogenesis can contribute to the receipt of oxygen or nutrients by injured cells or tissues. The sites of oxygen consumption and ATP generation are the mitochondria (Thomas and Ashcroft 2019). Mitochondria are beneficial for the regulation of cell proliferation and migration, even in response to hypoxia (Reichard and Asosingh 2019). They also play a decisive role as mediators of the apoptosis pathway (Nie et al., 2019).

There are two essential apoptotic signaling pathways: the death receptor–mediated pathway (extrinsic) and the mitochondrial-dependent pathway (intrinsic). Mitochondria play a pivotal role in apoptotic induction (Nie et al., 2019), and changes in the mitochondrial membrane potential (MMP) and Bcl-2 family proteins play an important role in the regulation of mitochondrial apoptosis. Studies have shown that the decrease in MMP and the involvement of Bcl-2 protein result in the release of cytochrome c (Cyt-c) from the mitochondria to the cytoplasm, which then further activates cysteine-aspartic proteases (caspases) (Wang et al., 2019). Caspase-3 is also called the death protease because it can cause chromatin shrinkage, DNA fragmentation, cell lysis, and apoptosis.

Additionally, it has been reported that LDH may be localized to mammalian mitochondria (Fulghum et al., 2019). SOD in mitochondria works as an important antioxidant enzyme against oxidative stress, and MDA affects the mitochondrial respiratory chain (Shen et al., 2016; Tong et al., 2018). Furthermore, LDH, SOD, and MDA are associated with cell injury, and SOD and MDA are also biomarkers of oxidative stress (Zhu et al., 2019). In our findings, LDH and MDA levels were significantly increased compared with those in normal cells, indicating injury aggravation. After GHI administration, LDH and MDA levels decreased. The activity of SOD decreased in the OGD group compared with that in normal cells, and after GHI administration, the SOD activity increased. This demonstrated that GHI administration may prevent proliferating cells from injury in mitochondria.

In addition, we also found that GHI administration effectively increased the expression of PI3K/Akt-associated protein in OGD-injured rBMECs. The PI3K/Akt pathway promotes neuroprotective and antiapoptotic effects. However, LY 249002, a PI3K inhibitor, blocked the efficacy of GHI, which revealed that a GHI-mediated antiapoptotic effect may be achieved *via* the PI3K/Akt pathway. Similar to the previous study, there is evidence that blocking the PI3K activity can reduce the expression of p-Akt in cells (Wu et al., 2020).

**FIGURE 16:**
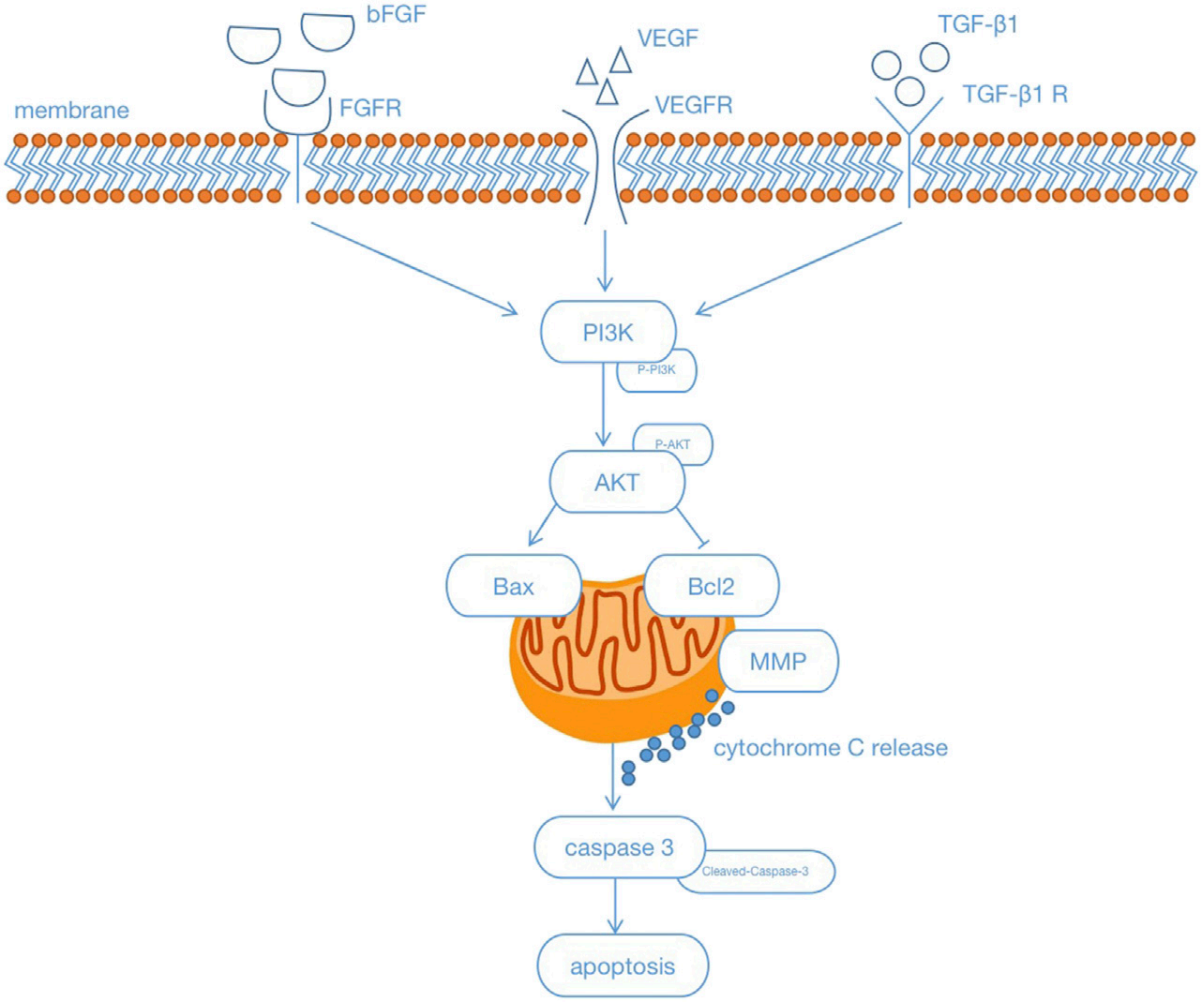
Schematic illustration showing that Guhong injection protected antiapoptosis and the integrity of the cerebral microvasculature and mitochondria through the PI3K/Akt signaling pathways under cerebral ischemia.

In summary, we conclude that treatment with GHI can protect brain tissue or rBMECs against ischemia-reperfusion injury or OGD-induced injury *via* repairing the cerebral microvasculature and mitochondria, and inhibiting apoptosis *via* activating the PI3K/Akt pathway under cerebral ischemia. Based on our findings, we provide a reference for clinical treatment with GHI (Figure 16). Thus, GHI may be an effective drug for treating cerebral ischemia because it assists in maintaining antiapoptosis, the cerebral microvasculature, and mitochondrial integrity.

## Conclusion

Our research initially proved that GHI exerted a distinct protective effect against cerebral I/R injury in rats and decreased rBMEC apoptosis during OGD injury. These protective effects were associated with its promotion of antiapoptosis and the integrity of the cerebral microvasculature and mitochondria through PI3K/Akt signaling pathways. Our obtained findings provide a reference for proper application of the multi-target GHI and can serve as a therapeutic option in the setting of brain syndrome.

## Data Availability

The original contributions presented in the study are included in the article/Supplementary Material; further inquiries can be directed to the corresponding authors.
