# Supplementary figures and images for: Guhong Injection Protects Against Apoptosis in Cerebral Ischemia by Maintaining Cerebral Microvasculature and Mitochondrial Integrity Through the PI3K/AKT Pathway
Source: Front Pharmacol. 2021 May 13;12:650983. doi: 10.3389/fphar.2021.650983 (PMC8155598; doi:10.3389/fphar.2021.650983)

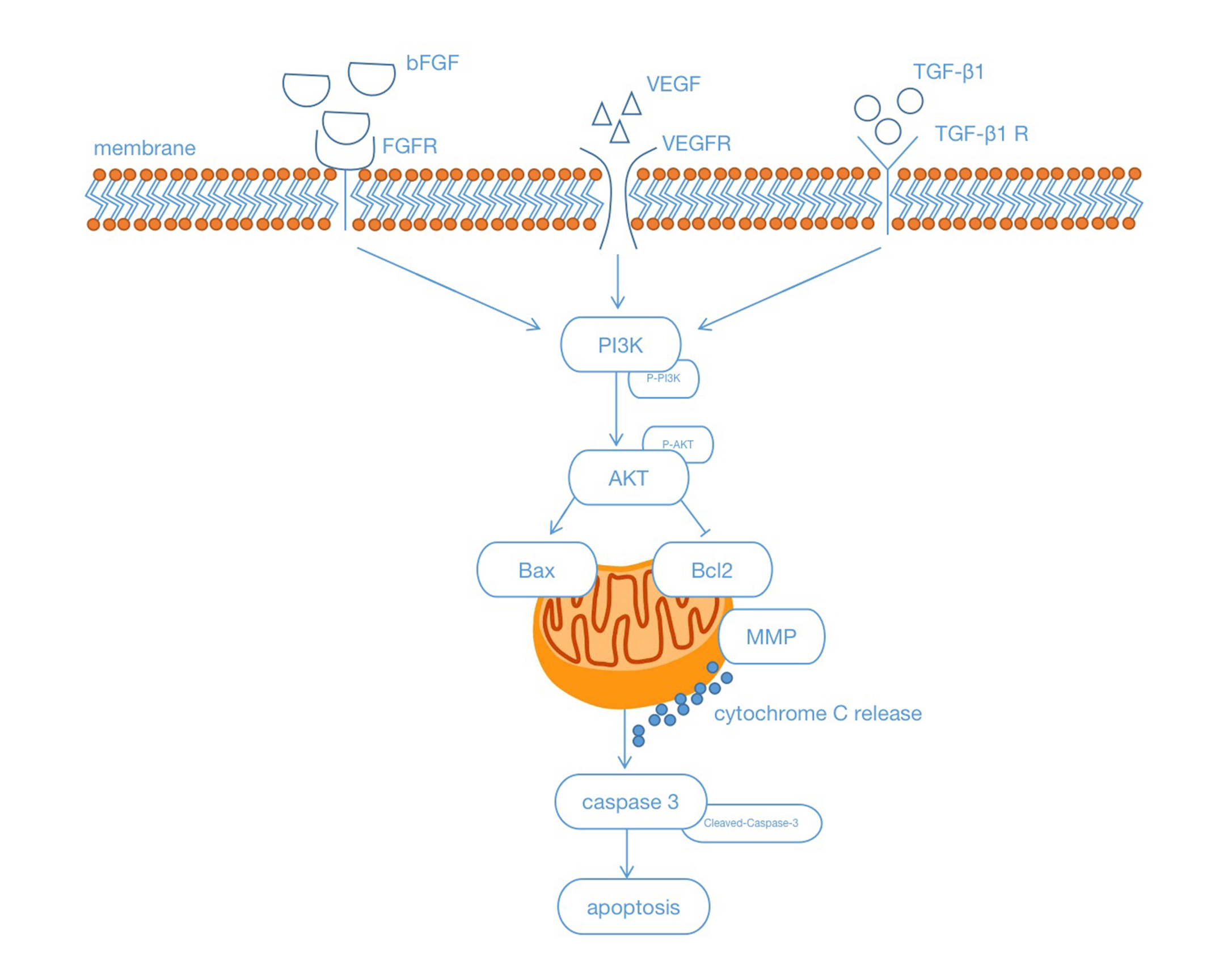

Supplement: Supplementary file 1 [file image2.jpeg]
