# Supplementary material for: Guhong Injection Protects Against Apoptosis in Cerebral Ischemia by Maintaining Cerebral Microvasculature and Mitochondrial Integrity Through the PI3K/AKT Pathway
Source: Front Pharmacol. 2021 May 13;12:650983. doi: 10.3389/fphar.2021.650983 (PMC8155598; doi:10.3389/fphar.2021.650983)
Supplement: Supplementary file 2 [file table1.doc]

**Supplementary table 1.** The MTT results of GHI in rBMECs after 6 h incubation (n=8)

| GHI Concentrations (μL/mL) | Cell viability (%) |
| --- | --- |
| 0 | 100.4 ± 1.2 |
| 6.25 | 105.1 ± 5.4 |
| 12.50 | 111.8 ± 6.5 |
| 25 | 114.3 ± 8.3 |
| 50 | 119.8 ± 6.5 |
| 100 | 126.5 ± 6.6 |
| 110 | 110.2 ± 6.9 |
| 120 | 82.3 ± 9.9 |
