# Supplementary material for: Guhong Injection Protects Against Apoptosis in Cerebral Ischemia by Maintaining Cerebral Microvasculature and Mitochondrial Integrity Through the PI3K/AKT Pathway
Source: Front Pharmacol. 2021 May 13;12:650983. doi: 10.3389/fphar.2021.650983 (PMC8155598; doi:10.3389/fphar.2021.650983)
Supplement: Supplementary file 3 [file image1.pdf]

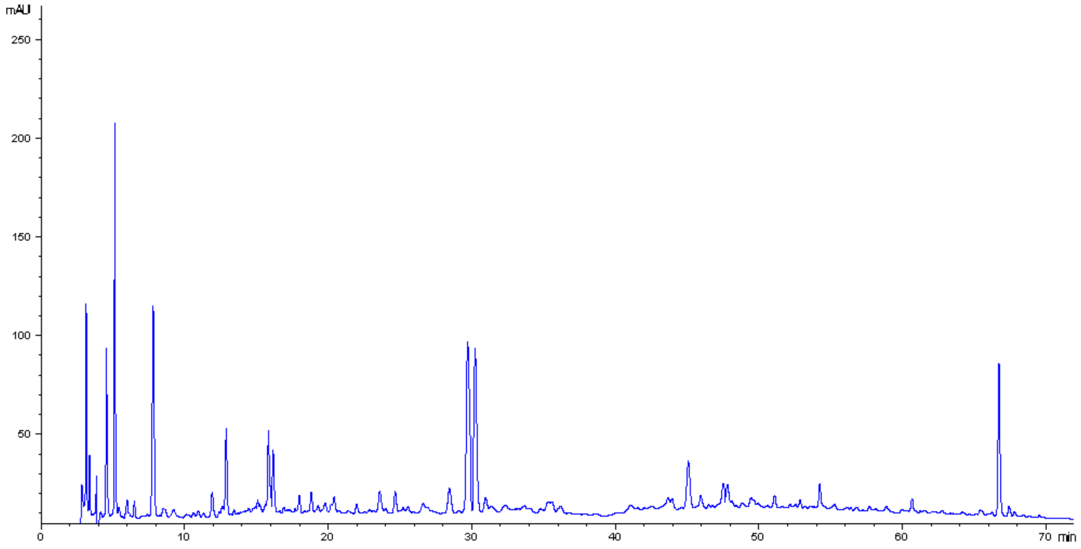

Supplementary figure 1 Chromatogram of Guhong injection (GHI) by injection by HPLC analysis at the UV detection wavelength of 280 nm.
